# Validation and determining an optimal cut‐off score of the Infant Behavior Checklist for autism spectrum disorder

**DOI:** 10.1002/pcn5.212

**Published:** 2024-06-18

**Authors:** Toshinobu Takeda, Hirokazu Osada, Yui Tsuji, Hiroshi Kurita

**Affiliations:** ^1^ Faculty of Psychology Ryukoku University Kyoto Japan; ^2^ Teikyo Heisei University Tokyo Japan; ^3^ Sapporo Gakuin University Sapporo Japan; ^4^ Zenkoku Ryouiku Soudan Center Tokyo Japan

**Keywords:** autism spectrum disorder, Japan, questionnaire, screening, validation

## Abstract

**Aim:**

Given the escalating prevalence of autism spectrum disorder (ASD), the persistent paucity of child psychiatrists in Japan, and the need to prepare for unforeseen situations, such as the COVID‐19 pandemic, it is essential to establish a reliable screening tool. This study aims to validate the Infant Behavior Checklist (IBC) developed by Japanese experts and determine its appropriate cut‐off score.

**Methods:**

A total of 354 clinic‐referred children, along with their caregivers, participated in this research. Clinical records, including diagnoses established through the sub‐structured diagnostic interviews, and the IBC assessments, were subjected to rigorous statistical analysis.

**Results:**

Among the 24 items, six failed to reach significance to differentiate ASD from non‐ASD cases. The Cronbach's alpha coefficient for the IBC was calculated at 0.77. The IBC total score for ASD cases was significantly higher than that of non‐ASD cases. With the chosen cut‐off score, the IBC demonstrated an area under the ROC curve of 0.803, along with sensitivity, specificity, positive predictive value, and negative predictive value of 8.03, 0.79, 0.69, 0.34, and 0.94, respectively.

**Conclusion:**

The IBC exhibits satisfactory internal consistency and content and discriminant validity. The high sensitivity and other associated indices for the optimal cut‐off score of the IBC affirm its validity as a screening instrument for ASD. Nevertheless, further investigations are warranted to ascertain the clinical utility of the IBC.

## INTRODUCTION

Autism spectrum disorder is characterized by persistent deficits in social communication and interaction and the presence of restricted and repetitive behaviors and interests Diagnostic and Statistical Manual of Mental Disorders (DSM‐5).^1^ The global prevalence of ASD is around 100 per 10,000 with a 4.2 male‐to‐female ratio and 33.0% of co‐occurring intellectual disability.^2^ ASD can be a costly disorder across one's lifetime. Early intervention is vital to teach skills across a variety of domains and prevent the development or exacerbation of behavioral deficits and excesses, considering early brain plasticity in young children.^3^

Currently, there is no medical treatment to completely cure the ASD condition, and nonpharmacological early interventions, including behavioral, developmental, naturalistic developmental behavioral, treatment and education of autistic and related communication handicapped children, sensory‐based, animal‐assisted, and technology‐based interventions constitute the mainstream methods for addressing ASD in children, albeit with some scholarly skepticism concerning their efficacy.^4^

A diagnosis of ASD may be made as early as 18–24 months of age when the aforementioned autistic traits emerge. Among the myriad of clinical techniques for evaluating individuals with ASD, the most prominent include the Autism Diagnostic Interview‐Revised, Autism Diagnostic Observation Schedule, Childhood Autism Rating Scale, and the Pervasive Developmental Disorders Autism Society Japan Rating Scale in Japan.^5^ Although they have been clinically proven to be effective instruments in differentiating autism from other related developmental disorders, they have been criticized for their time‐consuming nature, extensive questionnaires and intricate scoring methods, and the imperative requirement of licensed clinicians and observers for their administration.^6^

The advent of the COVID‐19 pandemic exacerbated the challenges associated with ASD detection in children. Throughout this period, medical or psychological practitioners confronted formidable barriers to conducting developmental assessments or even direct observations of children chiefly due to contagion risk (i.e., maintaining physical distance). In this globalized world, there is no warranty that a worldwide pandemic might not occur again in the near future. The method to differentiate and treat children with ASD should be established to prepare for this kind of contingency.

There is a chronic paucity of child psychiatrists and other allied professionals in Japan and this compels the development of efficient tools to differentiate children with ASD.^7^ Healthcare practitioners do not have enough time to see or administer questionnaires or tests to all children with a developmental delay, which is the most frequent chief complaint among preschool‐aged children with ASD. Therefore, children preliminarily suspected of ASD should receive expedited assessment.

Beyond conventional clinical diagnostic methodologies, certain self‐administered screening instruments tailored for toddlers and children have been devised and one of them is available in Japan. The Japanese version of the Modified Checklist for Autism in Toddlers (M‐CHAT) consists of 23 yes–no format items with satisfactory sensitivity and specificity. It was proven to be useful even for community samples.^8^, ^9^ However, it can only be used to detect autism in toddlers aged between 18 and 24 months. Considering the higher rate of high‐functioning ASD (i.e., IQ ≥ 70), the characteristics of ASD often emerge after the toddler years have elapsed.

In the light of the influential role that culture plays in shaping expectations for and demonstration of social communication and interaction skills,^10^ a screening tool should ideally be developed by experts within the local milieu. In this study, an original screening questionnaire created by experienced Japanese practitioners is validated, and its cutoff score is set for clinical application.

## METHODS

### Participants

Candidates for this study were 496 children who visited the X child development support center in Tokyo for the first time with the main chief complaints of developmental delay from 2011 to 2018. The opt‐out letters, including a clear notification covering the voluntary nature of participation, research objectives, the criteria for selecting prospective participants, personal information to be used in the study, confidentiality assurances, and the right to refuse participation, were sent to their caregivers. Twenty‐two candidates declared that they did not want to participate in this study. Sixty‐one letters were returned probably because of moving away. From the rest of the 413 children, 59 children whose IBC had three or more missing values (i.e., more than 10% of all the questions) were omitted. The missing values of 82 children whose IBC had one or two missing values were complemented by the SPSS missing value software (the expectation‐maximization algorithm). Consequently, 354 participants remained in this study (264 males and 90 females).

The children had been medically diagnosed with ASD or non‐ASD by the sub‐structured ASD diagnostic interview at an average of 32.10 (±34.90) days ranging from 1 to 252 days after caregivers had filled out the IBC. Their average age at the first visit was 45.3 (±14.19) months (ranging from 15 to 72 months). At the first visit, 213 and 141 children were administered the K‐Test and the Tanaka–Binet Test (the Japanese version of the Stanford–Binet Test [B‐test]), respectively.

### Tools

#### Infant Behavior Checklist

The Infant Behavior Checklist (IBC) was conceptualized during the infant behavior assessment workshop, with the primary aim of capturing the nascent indication of autism in the 1990s. Drawing upon their collective expertise and extensive review of extant literature, seasoned child psychiatrists and psychologists, including Dr. Naruse, the workshop's principal representative, meticulously curated a roster of 24 elements, thereby constituting a parent‐rating questionnaire.

These 24 elements are dichotomously segmented into two distinct categories: Items 1 through 12 encompass aberrant behaviors commonly observed within the initial years of life, while Items 13 through 24 pertain to those typically manifesting during the second and third years. Caregivers are enjoined to respond to each query employing a binary “yes–no” modality, discerning their child's developmental and behavioral attributes. For instance, caregivers would indicate “Yes” for Item 1, “Don't smile or gaze at parents when dandled,” should they discern an absence of such social gestures in their child.

Notably, it is imperative to underscore that, as the IBC examines early infantile behaviors, assessments should exclusively encompass the period preceding the child's third birthday. Consequently, if a child is older than 3 years, caregivers must mark “No” for behaviors covered by relevant items, even if such behaviors are currently observable but were previously absent prior to the age of 3 years.

The summative scoring methodology entails the aggregation of affirmative responses across the spectrum of 24 inquiries answered in a yes or no format. Ergo, the IBC's aggregate score spans a range from 0 to 24.^11^

The original version of the IBC (in Japanese) can be obtained by contacting the corresponding author.

#### The Pervasive Developmental Disorders Assessment System

The Pervasive Developmental Disorders Assessment System (PDDAS) is a Japanese semi‐structured interview system consisting of 91 items, including 12 major items corresponding to 12 items in criterion A of the DSM‐IV autistic disorder criteria; 36 items on autistic symptoms, and three Asperger's disorder (AS) screening items for diagnosing pervasive developmental disorders (PDD) and their subtypes; and 40 items for other information, including early development and past/family histories. The PDDAS was administered to mothers of 77 PDD children and 64 non‐PDD children. As a result, the PDDAS had satisfactory interrater reliability (ranges of k, r, and raw agreement rate were 0.69–1.00 in 76 items, 1.00 in 11 items, and 0.91–1.00 in four k un‐calculable items, respectively). Thirty‐three of the 36 items and all of the 12 major items scored significantly higher in the PDD than the non‐PDD groups to show satisfactory discriminant validity. PDDAS and consensus DSM‐IV diagnoses agreed in the 77 children with PDD diagnosis and disagreed in only two children in subtype diagnoses of autistic disorder and PDD not otherwise specified. The PDDAS, which takes 1.5 h to administer, has clinical and research utility.^12^ According to the DSM‐IV, the PDDAS can divide PDD into three categories: autistic disorder, AS, and PDD not otherwise specified; however, in this study, these three were collapsed into ASD following DSM‐5. In this study, the PDDAS was administered solely by a psychiatrist.

#### The Kyoto Scale of Psychological Development

The Kyoto Scale of Psychological Development (K‐Test) is a widely used developmental test standardized for 1562 0‐ to 13‐year‐old Japanese children, with satisfactory reliability and validity. It is also frequently used to assess the development of mentally handicapped infants and young children in Japan.^13^ The K‐Test consists of three subtests: posture‐movement (P‐M), cognition‐adaptation (C‐A), and language‐sociability (L‐S). The P‐M subtest consists of two items: standing and footstep.

The C‐A subtest consists of 10 items: toy blocks, block design, task box/square composition, little bell, puzzle/figure discrimination, puzzle/folding paper, drawing, figure drawing, nesting cup/weight comparison, and cups/memory/hitting blocks. The L‐S subtest consists of nine items: digit span, counting fingers, counting/calculation, selecting numbers, comparison, pointing by finger/vocabulary/naming, pointing figures/name/omission, gender/right‐and‐left discrimination, and comprehension/word definition.

In each subtest, a score is converted to developmental age (DA), and full‐scale DA is obtained. Developmental quotients (DQ) (full‐scale DQ, P‐M DQ, C‐A DQ, and L‐S DQ) are calculated according to rounding off the ratio of each DA to chronological age (CA) to the nearest whole number. If the B‐Test is difficult or even impossible to administer to the children, the K‐Test can be administered to them. The K‐Test is typically administered to infants or toddlers throughout Japan, except for the western region. Once children reach a mental age of 2 and a half years, the B‐Test is then employed for intellectual assessment. In this study, the K‐Test and B‐Test were employed on 213 and 141 children, respectively.

Koyama et al.^14^ showed the overall developmental quotient (DQ) of the K‐Test had a high correlation (Pearson's r, 0.88) with B‐Test IQ in children with ASD.

### Statistical analysis

First, to investigate how an answer to each IBC question predicts ASD diagnosis, the odds ratio (OR) for each question was calculated. Then, according to the results of the ORs, the revised short version of the IBC (IBC‐R) was made. The reliability of the IBC‐R is probed by calculating the indicator of internal consistency, Cronbach's α. At the same time, the IBC‐R scores of the two groups were compared by *t*‐test. Additionally, to study the validity of the IBS as a screening tool for ASD, the cut‐off score was set by the IBS‐R score. Data analyses were conducted in IBM SPSS 28 for Windows, with the threshold for statistical significance set at *p* < 0.05 (two‐tailed test).

This study was conducted with the approval of the review board at Ryukoku University (the ethics approval number is 2018–17; the date of approval is November 27, 2018). For this type of study, formal consent is not required.

## RESULTS

Out of 354 children, 58 cases were diagnosed with ASD by the PDDAS. The mean DQ/IQ of the ASD and non‐ASD groups were 69.43 (24.51) and 88.81 (19.01), respectively (*df* = 352, *t* = 6.73, *p* = 0.000). Table 1 shows the odds ratio of each item in the IBC in terms of ASD diagnosis. The data from non‐ASD cases is utilized as a reference. They range from 0.98 to 21.85. Out of 24 items, the lower limits of 95% confidence interval of six items (Items 2, 3, 5, 10, 17, and 22) could not reach 1 or over. In respect of screening efficacy, these six items were deleted, and the IBC‐R consisting of the remaining 18 items was formed.

**Table 1:** The odds ratio for each item within the Infant Behavior Checklist.

Item #	Contents	Percentage of positive responses		OR	95% CI	
		ASD (*n* = 58)	Non‐ASD (*n* = 296)		Lower	Upper
1	Lack of social smiling	6.89	0.34	21.85*	2.40	199.28
2	Hypersensitivity to sound	31.03	31.41	0.98	0.56	1.80
3	Hyposensitivity to sound	13.79	7.09	2.10	0.88	4.99
4	Reduced babbling	41.38	18.58	3.09*	1.70	5.63
5	Lack of stranger anxiety	43.10	34.80	1.42	0.80	2.52
6	Aloneness or indifference	37.93	18.58	2.68*	1.46	4.91
7	Lack of following	27.59	13.85	2.37*	1.22	4.60
8	No response to calling	46.55	12.16	6.29*	3.38	11.72
9	Poor facial expression	18.97	4.39	5.10*	2.16	12.04
10	No response to peek‐a‐boo	5.17	1.35	3.98	0.87	18.29
11	Lack of anticipatory motor adjustment	17.24	4.73	4.20*	1.76	9.99
12	Poor eye‐to‐eye contact	34.48	9.46	5.04*	2.59	9.81
13	Lack of pointing by fingers	46.55	15.20	4.86*	2.65	8.90
14	Speech delay (after 1.5 years old)	81.03	55.74	3.39*	1.69	6.80
15	Loss of meaningful words	18.97	7.77	2.78*	1.27	6.07
16	Lack of imitation	34.48	10.47	4.50*	2.33	8.68
17	Autostimulation behaviors	22.41	12.83	1.96	0.97	3.97
18	Extreme withdrawal	55.17	17.23	5.91*	3.25	10.76
19	Dislike being interrupted while playing	36.20	21.62	2.06*	1.13	3.76
20	No make‐believe play	56.90	23.31	4.34*	2.42	7.80
21	Repetitive behaviors	58.62	27.36	3.76*	2.10	6.73
22	Hyperactivity	58.62	47.64	1.56	0.88	2.75
23	Laughing/crying without any apparent reason	18.97	7.43	2.91*	1.33	6.40
24	Irregular and disturbed nocturnal sleep	31.03	18.92	1.93*	1.03	3.61

Abbreviations: ASD, autism spectrum disorder; OR, odds ratio; CI, confidence interval.

* *p* < 0.05.

Cronbach's alphas for the IBC and the IBC‐R are 0.77 and 0.76, respectively. The mean IBC scores for ASD and non‐ASD are 4.32 (3.28) and 8.63 (4.03), respectively. The mean IBC‐R scores for ASD and non‐ASD are 2.91 (2.72) and 6.69 (3.53), respectively. The IBC and the IBC‐R score for ASD is significantly higher than that of non‐ASD (*df* = 352, *t* = 8.79, *p* = 0.000 and *df* = 352, *t* = 9.20, *p* = 0.000, respectively). Cohen's *d* for the IBC and the IBC‐R are 1.17 and 1.20, respectively.

**Figure 1:**
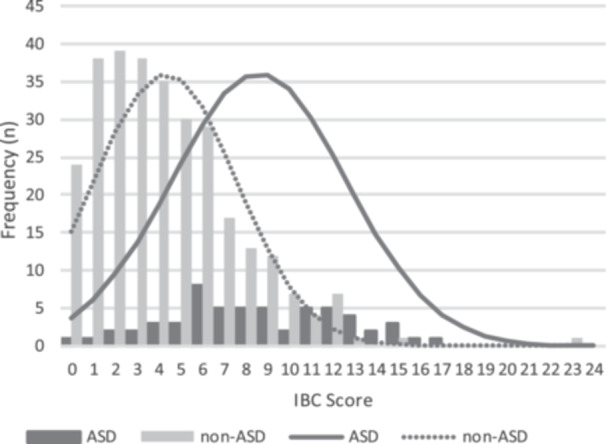
Distribution of Infant Behavior Checklist (IBC) scores by group. To facilitate a comparison between the autism spectrum disorder (ASD) curve and the non‐ASD curve, the peaks of both curves are aligned to be equal.

As for the cut‐off score, sensitivity, specificity, positive predictive value, and negative predictive value for the IBC and the IBC‐R are shown in Table 2. The area under the ROC curve for the IBC and the IBC‐R are 0.803 and 0.803, respectively. Figure 1 illustrates the distribution curve of the IBC score across different groups. According to Table 2, optimal cut‐off scores for the IBC and the IBC‐R are 6 and 5, respectively.

**Table 2:** Optimal cut‐off scores for the IBC and the IBC‐R.

IBC score	Sensitivity	Specificity	PPV	NPV
5	0.84	0.60	0.29	0.95
6	0.79	0.69	0.34	0.94
7	0.65	0.78	0.38	0.92

IBC‐R score	Sensitivity	Specificity	PPV	NPV
4	0.79	0.68	0.33	0.94
5	0.74	0.75	0.36	0.94
6	0.60	0.85	0.48	0.90

Abbreviations: IBC, Infant Behavior Checklist; IBC‐R, the revised short version of the IBC; NPV, negative predictive value; PPV, positive predictive value.

## DISCUSSION

This investigation has delineated the efficacy of the IBC as a screening tool for ASD. Both the IBC‐R as well as the conventional IBC have acceptable internal consistency. Since the IBC was developed by clinicians, including experienced child psychiatrists, its content validity could be reasonably conferred. Moreover, the ASD cohort scored significantly higher than their non‐ASD counterparts in both the IBC and the IBC‐R, thereby affirming the discriminant validity of these screeners.

With the advent of the DSM‐5 in 2013, the diagnostic criteria for ASD embraced hyper‐ or hyporeactivity to sensory stimuli. It is noteworthy that Japanese experts incorporated not only hyper‐ but also hyposensitivity to sound within the IBC in the 1990s. In this study, however, non‐ASD children exhibited sensory hyper‐ or hyposensitivity to sound (31% and 7%) at frequencies that did not significantly diverge from those observed in ASD children (31% and 14%). The results of this study doubted the uniqueness of hyper‐ or hyposensitivity in ASD since these symptoms can evenly occur in non‐ASD children with speech delay as well as children with ASD. Further inquiry is imperative to elucidate the intricate relationship between ASD and sensory responsiveness.

In comparison to the globally employed 23‐item M‐CHAT, which is widely used as an ASD screener all over the world, including in Japan, the IBC (24 items) shared as many as 10 items in common.

In the community‐based study of the Japanese version of the M‐CHAT, the IBC shared a substantial overlap of 10 items. In a community‐based study of the Japanese version of the M‐CHAT, the items most discriminative for 18‐month‐old children were “protoimperative pointing,” “imitation of action,” “pretend play,” “point following,” “language comprehension,” and “brings objects to show,” five items of which are related to preverbal social behaviors.^8^ Conversely, within this study, most discriminative items in the IBC are “lack of social smiling,” “no response to calling,” “extreme withdrawal,” “poor eye‐to‐eye contact,” “lack of pointing by fingers,” and “lack of imitation”; however, caution is needed since almost all our samples have chief complaints of speech delay (clinic‐based) and around half of the items differ from each other. Broadly, it appears the most discriminative items in the M‐CHAT and the IBC pertain to active and passive preverbal social behaviors, respectively.

The inclusion of “loss of meaningful words” as an item may be unique to the IBC. Dr. Kurita, one of the foremost authorities who developed the IBC, has studied similarities and differences between an autistic disorder (without speech loss), the “Knick” type of autism (characterized by speech loss), and childhood disintegrative disorder.^15^ In this study, 18% of the ASD group showed “loss of meaningful words” while 8% of the non‐ASD group did. Although childhood disintegrative disorder disappeared from the DSM‐5, developmental regression during childhood warrants continued investigation in the context of ASD.

Regarding the selection of the cut‐off scores, scores of 5 or 7 for the IBC and 4 or 6 for the IBC‐R are potential candidates as detailed in Table 2. Considering the figures of both sensitivity and specificity, especially giving precedence to sensitivity, the scores of 6 and 5 are set as the cut‐offs for the IBC and the IBC‐R, respectively. Sensitivity for each cut‐off surpasses 70.0%, which is regarded as clinically satisfactory. These results also indicate the validity of those scales as an ASD screener.

While an ideal screening tool should entail a parsimonious item set to minimize time consumption, judging from the results of this study, the IBC‐R, which deleted non‐significant items from the full IBC, did not enhance statistical metrics for the screening utility (i.e., sensitivity, specificity, positive predictive value, negative predictive value, and area under the curve). Importantly, these excluded items encompass clinically pertinent symptoms, such as hyper‐ or hyposensitivity to the sound and no response to peek‐a‐boo. Therefore, it would be prudent, for the time being, to employ the complete version of the IBC.

The IBC relies on parental recollection, which unavoidably associates shortcomings with the retrospective questionnaires. Furthermore, it is doubtful that parents would answer the IBC precisely remembering their child's behavior prior to the age of 3 years. For instance, it is plausible that parents may answer “yes” to questions even when relevant symptoms emerge after the age of 3 years.

This study found a notable gap in DQ/IQ levels between groups with and without ASD. Analyzing a birth cohort of 31,220 individuals, it was observed that almost half of those with ASD possessed an average or higher IQ. Conversely, nearly half of them exhibited either borderline IQ or intellectual disability.^16^ This prevalence appears to surpass that of the general population, potentially elucidating the significant contrast observed between the two groups in this study.

In clinical settings, it is often the case that IQ/DQ values are not yet known, and it is assumed that in many cases, IQ/DQ values cannot be considered as a simple screening tool. Therefore, in this study, we examined the utility of the screening scale without controlling for IQ/DQ values.^17^

The low specificity of 0.69 for the IBC could be a limitation of this study. Since ASD is considered to be a spectrum, it might be difficult to set a single cut‐off value in such tests.

In spite of several limitations, both the IBC and its abbreviated counterpart, the IBC‐R, originally conceived in Japan, exhibit notable utility as screening instruments. Further study is required with a more rigorous methodology and a larger sample size.

## AUTHOR CONTRIBUTIONS

Conception and design of the study: Hiroshi Kurita and Toshinobu Takeda. Acquisition and analysis of data: Hiroshi Kurita, Toshinobu Takeda, Yui Tsuji, and Hirokazu Osada. Drafting the manuscript or figures: Toshinobu Takeda, Yui Tsuji, and Hirokazu Osada. All authors contributed to and approved the final manuscript.

## CONFLICT OF INTEREST STATEMENT

The authors declare no conflicts of interest.

## ETHICS APPROVAL STATEMENT

The study was approved by the Ethics Committees of Ryukoku University. Participants were informed that their participation was voluntary.

## PATIENT CONSENT STATEMENT

Opt‐out notifications were dispatched to the guardians of all potential participants. In instances where guardians exercised their opt‐out preference or in cases where the notifications were returned, these particular cases were excluded from the inquiry.

## CLINICAL TRIAL REGISTRATION

N/A.

## Data Availability

All data generated or analyzed during this study are included in this published article.
